# Teduglutide in pediatric patients under 10 kg with short bowel syndrome on parenteral support: An open‐label study

**DOI:** 10.1111/ped.70301

**Published:** 2025-12-27

**Authors:** Kouji Masumoto, Mitsuru Muto, Takato Sasaki, Mitsuhiro Shikamura, Tomoko Tanaka, Sho Sakui, Masakazu Miyamoto, Hiroya Nakano, Taisuke Kondo, Satoshi Ieiri

**Affiliations:** ^1^ Department of Pediatric Surgery, Institute of Medicine University of Tsukuba Tsukuba Japan; ^2^ Department of Pediatric Surgery, Research Field in Medical and Health Sciences, Medical and Dental Area, Research and Education Assembly Kagoshima University Kagoshima Japan; ^3^ Takeda Pharmaceutical Company Limited Osaka Japan; ^4^ Takeda Pharmaceutical Company Limited Tokyo Japan

**Keywords:** intestinal failure, Japan, parenteral nutrition, pediatrics, short bowel syndrome

## Abstract

**Background:**

Patients with short bowel syndrome (SBS)‐associated intestinal failure (SBS‐IF) are dependent on parenteral support (PS). In Japan, teduglutide is the only GLP‐2 analog medicine indicated for these patients. There are limited data for pediatric patients, especially those with a low body weight. This study evaluated the safety and efficacy of teduglutide in Japanese pediatric patients with SBS‐IF (dependence on PS to provide ≥30% of fluid or caloric intake needs) who weighed less than 10 kg.

**Methods:**

This phase 3, open‐label study enrolled Japanese pediatric patients with SBS‐IF. Patients weighing less than 10 kg received teduglutide 0.05 mg/kg/day subcutaneously in 28‐week treatment cycles (24 weeks of teduglutide treatment and 4 weeks of follow‐up). Adverse events, changes in PS requirements, and growth parameters were assessed.

**Results:**

Three patients completed the study, with a mean teduglutide exposure duration of 48.9 weeks. All patients experienced treatment‐emergent adverse events (TEAEs), but none were related to the study drug or led to death or treatment discontinuation. Clinically meaningful reductions in mean PS volume (13.1%) and mean PS caloric intake (46.6%) were observed at the end of treatment from baseline. One patient achieved a reduction in PS volume of ≥20% at the end of treatment from baseline. Growth parameters showed increases in weight and height/length‐for‐age *z*‐scores at the end of treatment from baseline.

**Conclusion:**

In the three patients with SBS‐IF who weighed less than 10 kg, no new safety signals were observed following teduglutide treatment. Clinically meaningful reductions in PS were noted and there was no adverse impact on growth parameters.

**Trial Registration:**

ClinicalTrials.gov registration number: NCT05027308

## INTRODUCTION

Short bowel syndrome (SBS) is a congenital or acquired condition characterized by the loss of intestinal mass or function, which is generally a result of extensive resection of the bowel.^1^, ^2^, ^3^, ^4^ SBS is the leading cause of intestinal failure (IF) in children,^5^ in which the remnant intestine cannot support the nutritional and fluid requirements of the body.^3^, ^6^ The standard management of SBS‐associated IF (SBS‐IF) includes parenteral support (PS; parenteral nutrition and/or intravenous fluids).^7^, ^8^ Despite the administration of PS, pediatric patients with IF may continue to be underweight and experience stunted growth.^9^, ^10^ Moreover, long‐term PS may lead to fatal complications, such as IF‐associated liver disease (IFALD) and catheter‐related blood stream infections (CRBSIs).^4^, ^11^ Patients with SBS‐IF who have a low body weight for their age require careful monitoring to assist growth while avoiding these complications, which presents a critical clinical concern. Additionally, intestinal length increases rapidly during infancy and the early stages of childhood,^12^ making it essential to implement timely and efficient treatment strategies that promote intestinal adaptation to reduce PS dependency.^13^, ^14^

Glucagon‐like peptide‐2 (GLP‐2) is an enteroendocrine hormone that regulates the functional and structural integrity of the cells lining the gastrointestinal tract.^15^ Teduglutide is a recombinant analog of human GLP‐2, and has been shown to reduce PS dependence in adults and children with SBS‐IF.^16^, ^17^, ^18^, ^19^, ^20^, ^21^ Currently in Japan, teduglutide is the only approved GLP‐2 analog medicine available for patients with SBS‐IF. However, teduglutide data for patients who weigh less than 10 kg, including infants, are limited. This study aimed to evaluate the safety and efficacy of teduglutide in Japanese pediatric patients who weighed less than 10 kg with SBS‐IF (dependence on PS to provide ≥30% of fluid or caloric intake needs). The volume of teduglutide administered is based on body weight. For patients who weigh less than 10 kg, a 1.25 mg formulation of teduglutide (reconstituted to 2.5 mg/mL) has been developed and approved for use in Japan and the EU.^22^, ^23^ The teduglutide 1.25 mg formulation, which is used in this study, allows the precise administration of small volumes that are suitable for pediatric patients who weigh less than 10 kg (or <20 kg for patients with moderate‐to‐severe renal impairment).

## METHODS

### Trial design

This was a phase 3, open‐label study (ClinicalTrials.gov NCT05027308) that evaluated the teduglutide 1.25 mg formulation in Japanese pediatric patients with SBS‐IF, with a body weight less than 10 kg (or <20 kg if the patient had moderate‐to‐severe renal impairment). This study was conducted from January 2022 to September 2023, and in accordance with the International Council for Harmonisation Guideline for Good Clinical Practice and international and local regulatory standards. The study protocol and its amendments, informed consent documents, and recruitment materials were approved by the Institutional Review Board prior to study initiation. Patient informed consent was obtained from the patient and/or the patient's legal representative/parents and the investigator/investigator's delegates.

After a 2–4‐week screening period, eligible patients started a 28‐week treatment cycle, consisting of 24 weeks of teduglutide 0.05 mg/kg/day subcutaneous (SC) (or 0.025 mg/kg/day SC for patients with moderate‐to‐severe renal impairment) and a 4‐week follow‐up period (Figure S1).

Each patient visited the site at baseline, then weekly for the first 2 weeks (weeks 1 and 2), and every 4 weeks thereafter (weeks 4, 8, 12, 16, 20, 24, and 28).

Patients could escape the follow‐up period (weeks 24–28) and proceed immediately to another screening visit if they met one or more of the follow‐up period escape criteria after discontinuing teduglutide treatment: increased PS requirements, deteriorating nutritional status (such as body weight loss or growth failure) despite maximal tolerated enteral nutrition (EN), deteriorating fluid or electrolyte status despite maximal tolerated enteral fluid and electrolyte intake, severe diarrhea related to teduglutide discontinuation, and/or if the patient was omitted from a follow‐up period of a previous treatment cycle. Patients who escaped the follow‐up period and immediately proceeded to another screening visit started another cycle of teduglutide treatment if they met one or more of the treatment eligibility criteria and none of the exclusion criteria (Table S1). Otherwise, following completion of a 28‐week treatment cycle, patients could proceed to a no teduglutide treatment (NTT) period. During the NTT period, data were collected every 12 weeks and patients could proceed to another screening visit at any time if they met one or more of the treatment eligibility criteria. Treatment eligibility criteria included any of the follow‐up period escape criteria in addition to decreased PS requirements during teduglutide treatment but cessation of improvement after teduglutide discontinuation.

### Participants

Japanese pediatric patients with a corrected gestational age of 4 months or older with SBS‐IF (dependence on PS to provide ≥30% of fluid or caloric intake needs) were included in this study. At screening and baseline, patients had to weigh at least 5 kg and less than 10 kg if they had normal renal function or mild renal impairment (estimated glomerular filtration rate [eGFR] ≥50 mL/min/1.73 m^2^), or patients had to have a body weight of at least 10 kg and less than 20 kg if they had moderate‐to‐severe renal impairment (eGFR <50 mL/min/1.73 m^2^).

Key exclusion criteria were clinically significant intestinal obstruction, active or recurrent pancreatic/biliary disease, dysmotility that had prevented the advancement of enteral intake; intestinal malabsorption due to a genetic condition; severe dysmotility syndrome; major gastrointestinal surgical intervention, including significant intestinal resection or a bowel lengthening procedure within 3 months prior to screening; and a history of cancer or known cancer predisposition syndrome. Full inclusion and exclusion criteria are available in Table S1.

### Endpoints

Safety endpoints were evaluated using a range of parameters. Parameters included the incidence of treatment‐emergent adverse events (TEAEs), defined as adverse events that occurred or worsened in severity during or after receiving teduglutide. Serious adverse events (SAEs) and adverse events of special interest (AESIs) were also recorded, with an AESI defined as a TEAE (serious or non‐serious) of scientific and medical concern specific to teduglutide. AESIs included the growth of pre‐existing colon polyps; benign neoplasms within the gastrointestinal tract and the hepatobiliary system; and neoplasms with the potential to promote tumor growth, including both benign and malignant neoplasms not restricted to the gastrointestinal tract or to the hepatobiliary system. TEAEs and SAEs were coded according to the Medical Dictionary for Regulatory Activities (MedDRA version 26.0). SAEs were also summarized by severity and by relationship to the study drug. Physical examinations, gastrointestinal‐specific test results (including fecal occult blood testing and endoscopic evaluations), vital signs (including body temperature, respiratory rate, blood pressure, and pulse), and laboratory safety data (such as biochemistry, hematology, and urinalysis) were also assessed. Growth parameters were measured, including body weight‐for‐age and height or length‐for‐age *z*‐scores. In infants, weight‐for‐length *z*‐score was measured. Urine output was measured by recording urine volume for patients who were toilet‐trained and weighing urine‐only diapers for those not toilet‐trained. Stool output was assessed by bowel movements and stool consistency using the Bristol Stool Form Scale for patients who were toilet‐trained and weighing mixed (urine and stool) diapers for those not toilet‐trained.

Efficacy endpoints were assessed at each visit and at end of treatment (EOT) and included the change and percent change from baseline in PS volume, the proportion of patients achieving a ≥20% reduction in PS volume from baseline, and the proportion of patients achieving enteral autonomy (defined as complete weaning off PS). The change from baseline in the number of days per week of PS was recorded for each visit and at EOT. An intake diary was completed every day from screening until EOT or study termination. Output diaries recorded over 48‐h periods before scheduled visits yielded mean daily urine and fecal outputs, calculated relative to body weight. PS volume and infusion duration and EN volumes were also recorded. Actual PS and EN calories were calculated from diary entries. PS volume and PS caloric intake values reported in both patient intake diaries and in the electronic case report forms (eCRFs) with investigator‐prescribed data were normalized to body weight.

### Statistical analysis

Approximately five patients were planned for enrollment. The sample size was determined based on enrollment feasibility of this rare condition in Japanese pediatric patients, rather than by statistical power calculation. The study was not designed or sufficiently powered to determine the statistical significance of safety or efficacy endpoints.

Safety analyses were conducted using the safety analysis set, which included all patients who received at least one dose of teduglutide. The extent of exposure and extent of observation were calculated by treatment cycle and for the overall treatment period. Efficacy analyses were conducted using the full analysis set, which comprised patients who passed screening, irrespective of whether they received teduglutide.

Descriptive statistics were used for reporting data. Frequency and percentage were used to summarize TEAEs; mean and standard deviation (SD) were calculated for baseline characteristics, urine/fecal output, and treatment exposure/compliance; mean and standard error (SE) were calculated for PS volume, PS caloric intake, weight‐for‐age and height or length‐for‐age *z*‐scores, and number of days per week of PS use. Derived parameters, such as *z*‐scores for weight‐for‐age and height or length‐for‐age, were calculated using standardized formulas and reference data tables. The *z*‐scores for infants were based on corrected gestational ages.

## RESULTS

### Baseline characteristics

Three Japanese patients were enrolled in this study, including a 2‐year‐old child, a 1‐year‐old child, and an 11‐month‐old infant with a corrected gestational age of 10 months. Patient demographics and baseline characteristics are reported in Table 1.

**TABLE 1:** Demographics and baseline characteristics of individual patients.

Patient	Age	Primary etiology for SBS	Total estimated remaining small intestinal length^a^ (cm)	Estimated remaining colon (%)	Ileocecal valve present	PS treatment duration at the start of study (days)	On enteral nutrition	PS volume^b^ (mL/kg/day)	PS caloric intake^b^ (kcal/kg/day)	Weight‐for‐age *z*‐score^c^	Height or length‐for‐age *z*‐score^c^	ALT (U/L)	AST (U/L)
1	2 years (35 months)	Necrotizing enterocolitis	0	60	No	1064	No	125.3	69.0	−2.8	−1.9	113	86
2	1 year (21 months)	Necrotizing enterocolitis	60	80	No	503	Yes	72.7	59.3	−1.3	−3.1	71	52
3	11 months^d^	Intestinal torsion	80	100	Yes	352	Yes	73.5	48.1	−4.9	−4.2	141	99

Abbreviations: ALT, alanine aminotransferase; AST, aspartate aminotransferase; PS, parenteral support; SBS, short bowel syndrome.

^a^ The length of the jejunum and ileum below the ligament of Treitz.

^b^ Based on diary data.

^c^ *z*‐scores were calculated using the deviation of the value for an individual from the mean value of the reference population divided by the standard deviation for the reference population. The *z*‐score for the infant was based on the corrected gestational age.

^d^ Corrected gestational age was 10 months.

Two patients had necrotizing enterocolitis and one patient had intestinal torsion as their SBS‐IF etiology. The mean (SD) duration of SBS‐IF history was 18.4 (9.0) months. The mean (SD) estimated remaining small intestinal length was 46.7 (41.6) cm, with one patient having a distal/terminal ileum, in which an ileocecal valve was present. None of the patients had a stoma at baseline. The mean (SD) estimated percent of remaining colon was 80.0% (20.0), with all patients having colon‐in‐continuity.

All patients were dependent on PS, with two patients who also used EN. The mean (SD) duration of PS dependency was 2.1 (1.1) years for the two children and 11.3 months for the infant patient. The duration of EN usage was 1.7 years for the children and was 10.8 months for the infant. Patients had a mean (SE) PS volume of 90.5 (17.4) mL/kg/day and a mean (SE) PS caloric intake of 58.8 (6.0) kcal/kg/day at baseline. At baseline, the mean (SE) weight‐for‐age *z*‐score was −3.0 (1.0) and height or length‐for‐age *z*‐score was −3.1 (0.7). The weight‐for‐length *z*‐score for the infant patient was −4.0 at baseline.

All patients had concurrent medical conditions, with one having low birth weight, premature birth, functional gastrointestinal disorder, and liver disorder. For the other two patients, one experienced dermatitis diaper and the other had general dermatitis. Baseline mean (SD) urine output was 52.0 (11.6) mL/kg/day and mean (SD) fecal output was 64.7 (18.8) g/kg/day.

### Treatment exposure and compliance

None of the patients had moderate‐to‐severe renal impairment and all patients received teduglutide 0.05 mg/kg/day SC. The overall mean (SD) duration of teduglutide exposure was 48.9 (2.1) weeks and the overall duration of observation was 60.9 (13.8) weeks. The overall mean (SD) study treatment compliance was 99.9% (0.2), with all patients completing at least two treatment cycles. The last dose of teduglutide was administered in cycle 2, week 24 for patient 1, cycle 3, week 3 for patient 2, and cycle 2, week 24 for patient 3.

### Safety

All three patients experienced a total of 26 TEAEs (Table 2). Most TEAEs were mild or moderate in severity. Four severe TEAEs were reported in one patient, all of which were vascular device occlusion. None of the TEAEs were related to the study drug. Two patients had enterocolitis, COVID‐19, device‐related infection, and respiratory syncytial virus infection (Table 3).

**TABLE 2:** Overall TEAEs (safety analysis set).

TEAE	Teduglutide (*N* = 3)	
	*n* (%)	Number of events
Any TEAE^a^	3 (100.0)	26
Mild	3 (100.0)	11
Moderate	3 (100.0)	11
Severe	1 (33.3)	4
Any SAE	3 (100.0)	12
Mild	0	0
Moderate	3 (100.0)	8
Severe	1 (33.3)	4
AESIs^b^	0	0
TEAEs^a^ related to the study drug	0	0
TEAEs^a^ leading to treatment discontinuation	0	0
TEAEs^a^ leading to death	0	0

*Note*: Patients were counted only once for incidence but could be counted multiple times for the number of events. Patients with multiple relationships were only counted once in the ‘related’ incidence but could be counted multiple times in the number of events.

Abbreviations: AESI, adverse event of special interest; SAE, serious adverse event; TEAE, treatment‐emergent adverse event.

^a^ TEAEs were summarized by patient incidence and event count. In cases of missing severity data, TEAEs were classified as severe; if relationship data were missing then TEAEs were assumed to be related to the study drug.

^b^ An AESI is a TEAE (serious or non‐serious) of scientific and medical concern specific to teduglutide.

**TABLE 3:** TEAEs by system organ class and preferred term (safety analysis set).

TEAE	Teduglutide (*N* = 3)	
	*n* (%)	Number of events
Any TEAE	3 (100.0)	26
Gastrointestinal disorders	2 (66.7)	2
Enterocolitis	2 (66.7)	2
General disorders and administration site conditions	2 (66.7)	6
Catheter site pruritus^a^	1 (33.3)	1
Catheter site rash	1 (33.3)	1
Vascular device occlusion^a^	1 (33.3)	4
Hepatobiliary disorders	1 (33.3)	1
Liver disorder	1 (33.3)	1
Infections and infestations	3 (100.0)	12
COVID‐19	2 (66.7)	2
Device‐related infection^a^	2 (66.7)	5
Respiratory syncytial virus infection	2 (66.7)	2
Bacteremia^a^	1 (33.3)	1
Parainfluenza virus infection	1 (33.3)	1
Skin candida	1 (33.3)	1
Investigations	1 (33.3)	1
Blood iron increased	1 (33.3)	1
Metabolism and nutrition disorders	1 (33.3)	1
Lactic acidosis	1 (33.3)	1
Product issues	1 (33.3)	1
Device breakage^a^	1 (33.3)	1
Renal and urinary disorders	1 (33.3)	1
Oliguria	1 (33.3)	1
Respiratory, thoracic, and mediastinal disorders	1 (33.3)	1
Upper respiratory tract inflammation	1 (33.3)	1

*Note*: TEAEs were defined using MedDRA version 26.0.

Abbreviations: MedDRA, Medical Dictionary for Regulatory Activities; SAE, serious adverse event; TEAE, treatment‐emergent adverse event.

^a^ These were all SAEs.

Most TEAEs were reported once in individual patients. In total, 12 SAEs were reported in all patients. Five SAEs were device‐related infection and four were vascular device occlusion, which were reported in two patients and in one patient, respectively. The remaining three SAEs of bacteremia, catheter site pruritus, and device breakage were reported once in individual patients. All SAEs were resolved, and none were considered related to the study drug. There were no TEAEs leading to death, no TEAEs leading to treatment discontinuation, and no AESIs during the study. Patient 1 experienced a mild liver disorder on day 337 (alanine aminotransferase [ALT] and aspartate aminotransferase [AST] levels were 113 U/L and 86 U/L, respectively, at baseline and 44 U/L and 47 U/L, respectively, at week 24 of cycle 1, which increased to 391 U/L and 236 U/L, respectively, at week 24 of cycle 2), which was not considered related to the study drug.

During the study, no clinically meaningful changes were noted for any vital sign parameters. Similarly, no clinically meaningful changes were observed for hematology, biochemistry, or coagulation evaluations.

All patients consistently had diarrhea (types 6 and 7 on the Bristol Stool Form Scale) throughout the treatment cycles (loose stool [type 5] was reported at one visit in one patient). There were no clinically meaningful changes to urine or fecal outputs observed during the study. The mean (SD) daily urine output at EOT was 53.7 (17.6) mL/kg/day, which corresponded to a mean (SD) increase of 6.5% (44.0) from baseline. The mean (SD) daily stool/mixed stool diaper weight at EOT was 43.2 (23.4) g/kg/day, which corresponded to a decrease of 27.6% (40.7) from baseline.

### Changes in PS volume and PS caloric intake

**FIGURE 1:**
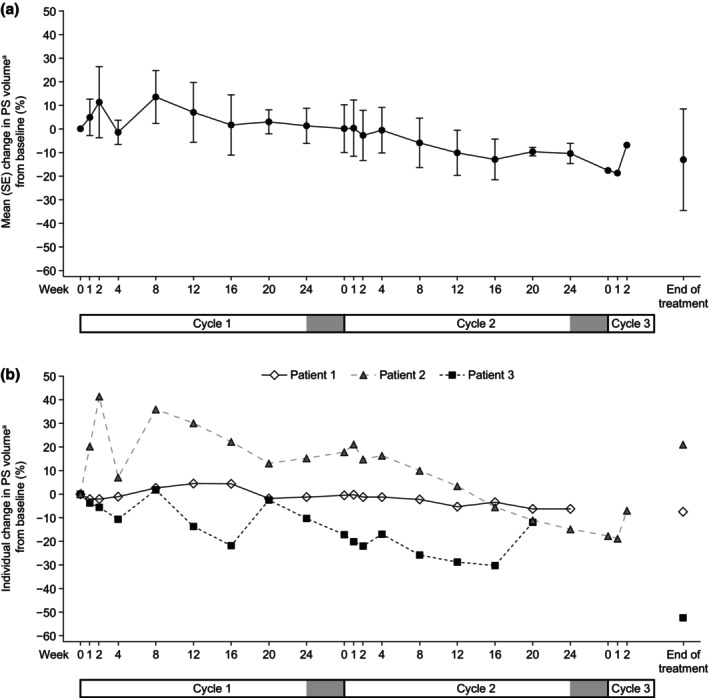
(a) Mean (SE) percent change in PS volume^a^ from baseline by visit. (b) Individual percent changes in PS volume^a^ from baseline by visit. The gray bars at the end of cycles 1 and 2 indicate no teduglutide treatment periods. ^a^Based on diary data. PS, parenteral support; SE, standard error.

The mean (SE) change in PS volume at EOT from baseline was −11.0 (15.6) mL/kg/day, which corresponded to a 13.1% (21.5) reduction in PS volume for the entire study (Figure 1). The mean (SE) changes in PS volume at the end of cycle 1 and at the end of cycle 2 from baseline were 0.7 (5.5) mL/kg/day and −9.1 (0.9) mL/kg/day, respectively, which corresponded to a mean (SE) increase of 1.2% (7.5) and a mean (SE) decrease of 11.0% (2.5), respectively.

**FIGURE 2:**
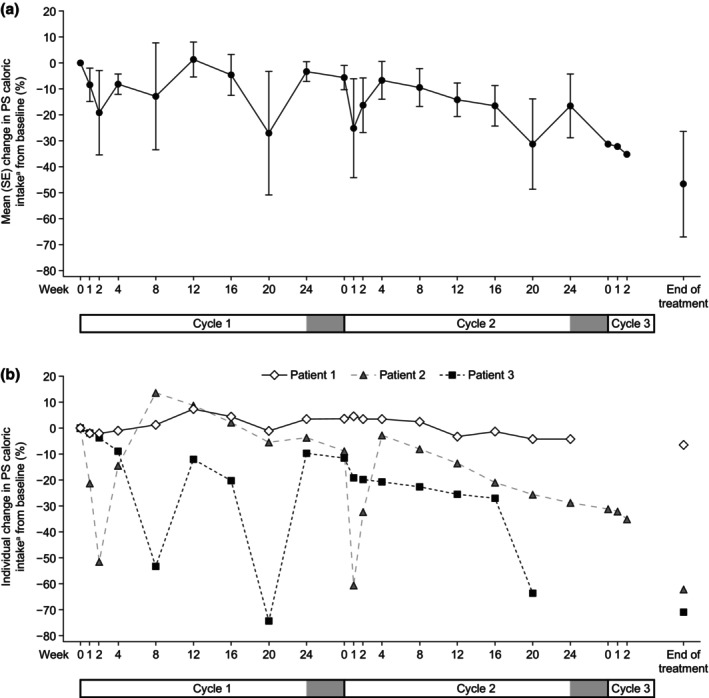
(a) Mean (SE) percent change in PS caloric^a^ intake from baseline by visit. (b) Individual percent changes in PS caloric intake^a^ from baseline by visit. The gray bars at the end of cycles 1 and 2 indicate no teduglutide treatment periods. ^a^Based on diary data. PS, parenteral support; SE, standard error.

The mean (SE) change in PS caloric intake at EOT from baseline was −25.2 (10.4) kcal/kg/day, which corresponded to a 46.6% (20.3) reduction in PS caloric intake for the entire study (Figure 2). The mean (SE) changes in PS caloric intake at the end of cycle 1 and at the end of cycle 2 from baseline were −1.5 (2.1) kcal/kg/day and −16.9 (8.0) kcal/kg/day, respectively, which corresponded to mean (SE) decreases of 3.3% (3.8) and 32.3% (17.2) respectively.

One patient achieved a ≥20% reduction in PS volume at EOT. None of the patients were weaned off PS during the study. There was no change in the mean (SE) number of days per week of PS use at EOT (7 [0] days) compared with baseline (7 [0] days) during teduglutide treatment.

### Changes in growth parameters

**FIGURE 3:**
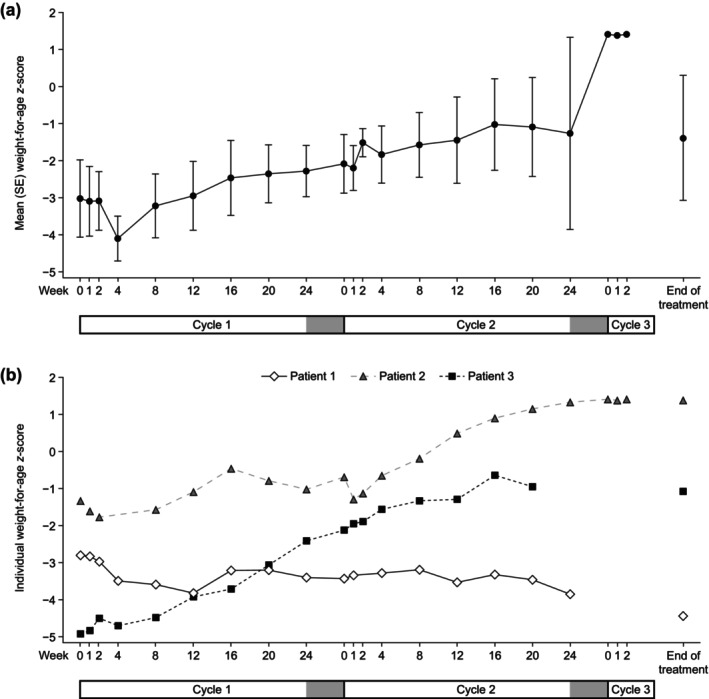
(a) Mean (SE) weight‐for‐age *z*‐score by visit. (b) Individual weight‐for‐age *z*‐score by visit. The gray bars at the end of cycles 1 and 2 indicate no teduglutide treatment periods. SE, standard error.

**FIGURE 4:**
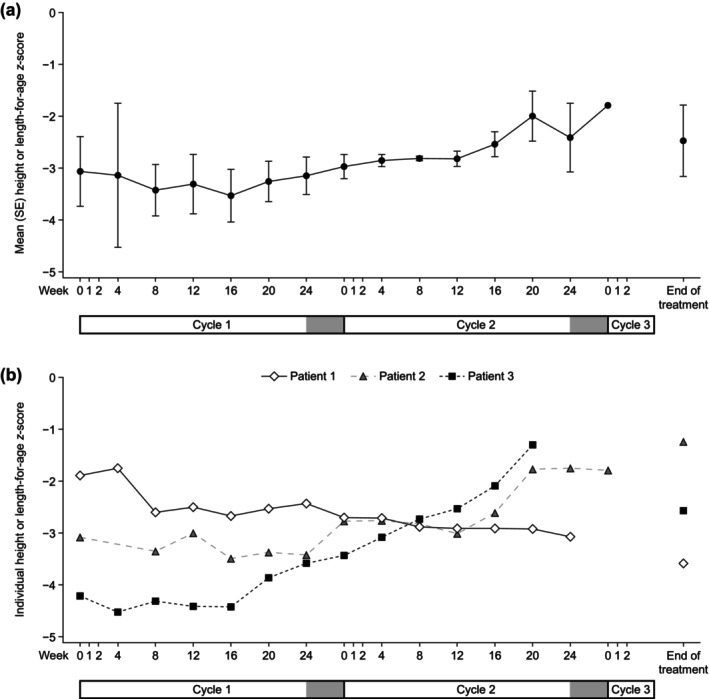
(a) Mean (SE) height or length‐for‐age *z*‐score by visit. (b) Individual height or length‐for‐age *z*‐score by visit. The gray bars at the end of cycles 1 and 2 indicate no teduglutide treatment periods. SE, standard error.

The mean (SE) changes from baseline to EOT for weight‐for‐age and height or length‐for‐age *z*‐scores were 1.6 (1.7) and 0.6 (1.2), respectively (Figures 3 and 4). The change from baseline to EOT for the weight‐for‐length *z*‐score for the infant patient was 4.2.

## DISCUSSION

In this open‐label study, patients treated with teduglutide demonstrated a safety profile consistent with previous pediatric studies, including both Japanese and non‐Japanese patients.^16^, ^17^, ^21^ No new safety signals or TEAEs considered related to the study drug were observed over 49 weeks in a small cohort of Japanese pediatric patients (including one infant) who weighed less than 10 kg and who had SBS‐IF. Despite the vulnerabilities inherent to this group, which included an increased risk of complications such as IFALD and CRBSI, teduglutide adherence remained high. This study also showed clinically meaningful reductions in PS volume and PS caloric intake without impeding growth. The three patients in this study had distinct clinical backgrounds and demonstrated different clinical courses and treatment responses to teduglutide.

We observed a decrease of 13.1% in PS volume and a decrease of 46.6% in PS caloric intake at EOT compared with baseline, with one patient experiencing a decrease of ≥ 20% in PS volume. Although no clinically meaningful changes in growth parameters were noted, there was an upward trend of growth improvement. The decrease in PS volume and PS caloric intake were greater at the end of cycle 2 than at the end of cycle 1 compared with baseline, which may suggest a delayed response to teduglutide, aligning with previous publications that have suggested that a longer duration of teduglutide treatment provides further clinical benefit.^16^ It has also been demonstrated that patients with SBS‐IF who have colon‐in‐continuity are more likely to benefit from longer treatment durations, with most patients achieving a clinically meaningful reduction in PS after 1 year of treatment.^24^

It is possible that despite receiving a high PS caloric intake, pediatric patients can still experience growth challenges.^25^ Additionally, prolonged high levels of PS caloric intake may lead to liver dysfunction,^26^, ^27^ which as a consequence can require a reduction in PS volume. These issues underscore the importance of promoting intestinal adaptation with teduglutide and managing PS‐related complications. In this study, two of the three patients (patients 2 and 3) had relatively long remaining small intestines and had poor growth despite PS administration at baseline. One of these patients (patient 3) also required reduced PS calorie intake owing to high levels of liver enzymes at baseline. Following teduglutide treatment, these patients experienced a decrease in PS caloric intake from baseline and also demonstrated improvements in growth parameters without any liver dysfunction. One patient (patient 2) required a transient increase in PS volume at EOT and this impacted the mean PS volume at EOT; however, this was considered to be related to a CRBSI in the preceding week. In addition, both patients achieved enteral autonomy after the trial (data not reported). These outcomes are consistent with preclinical data indicating that the administration of GLP‐2 at the appropriate timing can promote intestinal adaptation,^28^ suggesting that teduglutide may benefit pediatric patients with residual small intestines by further enhancing intestinal adaptation, even in patients with a low body weight.

Owing to a mild liver disorder, treatment interruption occurred at week 24 of cycle 2 in one patient (patient 1). The investigator deemed that the treatment interruption was related to the patient's worsening liver disorder, and not due to the study drug. This patient had a liver disorder at baseline and presented with premature birth and necrotizing enterocolitis, had no residual small intestine and 60% of remaining colon, and was highly dependent on PS. In Japan, small intestine transplantation is rarely performed, especially in patients under 10 kg, for whom the likelihood of identifying a suitable donor is particularly low. Therefore, treatment with teduglutide was used as a bridging strategy to support weight gain and maintain liver function while awaiting potential transplantation. To promote growth while protecting liver function in this patient, the total amount of PS was not substantially altered. However, as the patient gained weight gradually following teduglutide administration, the amount of PS per kilogram of body weight decreased. This reflects a cautious and individualized approach to balancing nutritional needs with the risk of liver dysfunction. Given the resection of the distal small intestine and the left half of the colon (i.e. sites that secrete GLP‐2), it is speculated that teduglutide facilitated adaptation of the colon.^29^ Importantly, no TEAEs related to teduglutide were observed in this patient. While the use of teduglutide in patients with no residual small intestine should be approached with caution, in this context, teduglutide provided a suitable option that may have contributed to stabilizing the patient's condition in the absence of other interventions.

In clinical settings, PS is carefully titrated for pediatric patients to ensure that energy needs for growth and development are met while optimizing intestinal adaptation with teduglutide treatment, and minimizing the risk of dehydration and electrolyte imbalances.^4^, ^30^ All patients in this study had a high fecal output before teduglutide administration, with consistent diarrhea and loose stools. Therefore, when considering reductions in PS volume, clinicians should be mindful of the risk of dehydration associated with these adjustments for patients already experiencing significant fluid loss. Moreover, during SBS‐IF nutritional treatment, clinicians should adjust the provision of lipid emulsions, carbohydrates, and amino acids, which can be associated with developing IFALD.^26^, ^27^ In this study, following teduglutide administration, patients had an overall decrease in PS caloric intake along with marginal improvements in growth parameters. Because of the clinically diverse backgrounds and unique management challenges of pediatric patients with SBS‐IF, in addition to the varied clinical benefits following teduglutide treatment, tailored treatment goals and strategies should be carefully considered to optimize clinical outcomes, particularly in pediatric patients with a low body weight for their age.

Limitations for this study include the small sample size and the open‐label design. However, with limited teduglutide data in a pediatric population who weigh less than 10 kg, our findings should offer valuable insights for both patients and physicians. Further studies are needed to evaluate long‐term outcomes and predictors of safety and efficacy when administering teduglutide to patients with SBS‐IF who weigh less than 10 kg in clinical practice.

In conclusion, no new safety signals were observed following treatment with teduglutide in the three Japanese pediatric patients with SBS‐IF who weighed less than 10 kg. The reported TEAEs in this study were generally consistent with the underlying disease and known adverse drug reactions previously reported in pediatric and adult patients who were treated with teduglutide. Together with clinically meaningful reductions in PS volume and PS caloric intake, teduglutide may provide another treatment option for pediatric patients with SBS‐IF who weigh less than 10 kg. Our data add to the existing literature, suggesting that teduglutide may be another treatment option for this pediatric patient population, along with careful adjustments to PS.

## AUTHOR CONTRIBUTIONS

K.M., M.Mu., T.S., S.S., M.Mi., H.N., T.K., and S.I. contributed to the acquisition, analysis, or interpretation of data. M.S. and T.T. contributed to the conception or design of the work. K.M., M.Mu., T.S., M.S., T.T., S.S., M.Mi., H.N., T.K., and S.I. critically revised the manuscript and gave final approval of the version to be published. All authors agree to be accountable for all aspects of the work ensuring integrity and accuracy.

## FUNDING INFORMATION

The study was funded by Takeda Pharmaceutical Company Limited, Tokyo, Japan.

## CONFLICT OF INTEREST STATEMENT

M.S., T.T., and M.Mi. are employees and stock owners of Takeda Pharmaceutical Company Limited. S.S., H.N., and T.K. are employees of Takeda Pharmaceutical Company Limited. K.M., M.Mu., T.S., and S.I. report no conflicts of interest.

## Supporting information


**Appendix S1:** Supporting Information.

## Data Availability

Takeda Pharmaceutical Company Limited does not plan to share the data supporting the results reported in this article because there is a reasonable likelihood that individual patients could be reidentified owing to the limited number of study participants.
