# Supplementary material for: Teduglutide in pediatric patients under 10 kg with short bowel syndrome on parenteral support: An open‐label study
Source: Pediatr Int. 2025 Dec 27;68(1):e70301. doi: 10.1111/ped.70301 (PMC12743259; doi:10.1111/ped.70301)
Supplement: Supplementary file 1 — Appendix S1: Supporting Information. [file PED-68-e70301-s001.docx]

**Teduglutide in pediatric patients under 10 kg with short bowel syndrome on parenteral support: An open-label study**

# SUPPLEMENTARY MATERIALS

TABLE S1 Full inclusion and exclusion criteria.

| Inclusion criteria: |
| --- |
| 1. In the opinion of the investigator, a parent/guardian had been capable of understanding and complying with protocol requirements. 2. A parent or guardian signed and dated a written, informed consent form and any required privacy authorization prior to the initiation of any study procedures. 3. Male or female pediatric patient of corrected gestational age 4 months or older. 4. Body weight at the time of screening and baseline visits of at least 5 kg and <10 kg for patients with normal renal function or mild renal impairment (eGFR ≥50 mL/min/1.73 m^2^), OR at least 10 kg and <20 kg for patients with moderate-to-severe renal impairment (eGFR <50 mL/min/1.73 m^2^). 5. Diagnosis of SBS with intestinal failure, defined as dependence on PS to provide at least 30% of fluid or caloric needed. 6. Patients to have stable PS for at least one month prior to screening as assessed by the investigator. Stable PS was defined as inability to significantly reduce PN/IV support, usually associated with minimal or no advance in enteral feeds (i.e. 10% or less change in PN or advance in feeds), assessed by the investigator. |
| Exclusion criteria: |
| 1. A parent/guardian who had been not capable of understanding or not willing to adhere to the study visit schedule and other protocol requirements. 2. Clinically significant intestinal obstruction, active or recurrent pancreatic or biliary disease, or dysmotility that had prevented the advancement of enteral intake. 3. Intestinal malabsorption due to a genetic condition, such as cystic fibrosis, microvillus inclusion disease, etc. 4. Severe, known dysmotility syndrome, such as pseudo-obstruction or persistent, severe, active gastroschisis-related dysmotility, that was the primary contributing factor to feeding intolerance and inability to reduce PS, prior to screening. Dysmotility was defined as severe if it was expected to limit the advancement of enteral feeding. 5. Major GI surgical intervention including significant intestinal resection or bowel lengthening procedure within 3 months prior to screening (insertion of feeding tube, anastomotic ulcer repair, minor intestinal resections ≤10 cm, and endoscopic procedures were allowed). 6. Cardiac disease that had made the patient vulnerable to changes in fluid status. 7. History of cancer or known cancer predisposition syndrome, such as juvenile polyposis or Beckwith–Wiedemann syndrome, or first degree relative with early onset of GI cancer (including hepatobiliary and pancreatic cancer). 8. Concurrent treatment with GLP-2, human growth hormone, or analogs of these hormones (not including teduglutide) within 6 months prior to the screening visit, or concurrent treatment with octreotide or GLP-1 analogs within 30 days prior to the screening visit. 9. Concurrent treatment with biological therapy (e.g. anti-TNF) for active Crohn’s disease within 6 months prior to the screening visit. 10. Participation in a clinical study using an experimental drug (other than glutamine or intravenous lipid emulsions) within 3 months or 5.5 half-lives of the experimental drug, whichever was longer, prior to the screening visit and for the duration of the study. 11. Known or suspected intolerance or hypersensitivity to the study drug, closely related compounds, or any of the stated ingredients. 12. Signs of active, severe, or unstable clinically significant hepatic impairment during the screening period as meeting at least two of any of the following parameters:     1. INR >1.5 not corrected with parenteral vitamin K     2. Platelet count <100×10^3^/μL due to portal hypertension     3. Presence of clinically significant gastric or esophageal varices     4. Cirrhosis     5. Persistent cholestasis defined as conjugated bilirubin >4 mg/dL (>68 μmol/L) over a 2-week period during screening     6. Total bilirubin ≥2× ULN     7. AST ≥3× ULN     8. ALT ≥3× ULN 13. Any condition, disease, illness, or circumstance that in the investigator’s opinion could put the subject at any undue risk, prevented completion of the study, or interferes with analysis of the study results. |

ALT, alanine aminotransferase; anti-TNF, anti-tumor necrosis factor; AST, aspartate aminotransferase; eGFR, estimated glomerular filtration rate; GI, gastrointestinal; GLP-1, glucagon-like peptide 1; GLP-2, glucagon-like peptide 2; INR, international normalized ratio; IV, intravenous; PN, parenteral nutrition; PS, parenteral support; SBS, short bowel syndrome; ULN, upper limit of normal.

FIGURE S1 Study design.


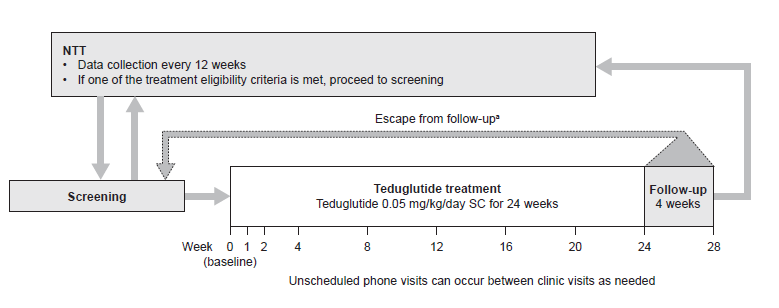


NTT, no teduglutide treatment; SC, subcutaneous.

^a^Patients could escape the follow-up period (weeks 24–28) and proceed immediately to another screening visit if they met one or more of the follow-up period escape criteria after discontinuing teduglutide treatment.
